# Synthesis, Structural Characterization, and Biological Activity Studies of Ni(II) and Zn(II) Complexes

**DOI:** 10.1155/2014/568741

**Published:** 2014-04-28

**Authors:** Palakuri Kavitha, K. Laxma Reddy

**Affiliations:** Department of Chemistry, National Institute of Technology, Warangal 506 004, India

## Abstract

Ni(II) and Zn(II) complexes were synthesized from tridentate 3-formyl chromone Schiff bases such as 3-((2-hydroxyphenylimino)methyl)-4H-chromen-4-one (HL_1_), 2-((4-oxo-4H-chromen-3-yl)methylneamino)benzoic acid (HL_2_), 3-((3-hydroxypyridin-2-ylimino)methyl)-4H-chromen-4-one (HL_3_), and 3-((2-mercaptophenylimino)methyl)-4H-chromen-4-one (HL_4_). All the complexes were characterized in the light of elemental analysis, molar conductance, FTIR, UV-VIS, magnetic, thermal, powder XRD, and SEM studies. The conductance and spectroscopic data suggested that, the ligands act as neutral and monobasic tridentate ligands and form octahedral complexes with general formula [M(L_1–4_)2]*·n*H_2_O (M = Ni(II) and Zn(II)). Metal complexes exhibited pronounced activity against tested bacteria and fungi strains compared to the ligands. In addition metal complexes displayed good antioxidant and moderate nematicidal activities. The cytotoxicity of ligands and their metal complexes have been evaluated by MTT assay. The DNA cleavage activity of the metal complexes was performed using agarose gel electrophoresis in the presence and absence of oxidant H_2_O_2_. All metal complexes showed significant nuclease activity in the presence of H_2_O_2_.

## 1. Introduction

Metal complexes of O, S, and N containing Schiff bases have been the subject of current and growing interest because it has wide range of pharmacological activities [1]. In particular Schiff bases with 2-amino thiophenol, 2-amino phenol, 2-amino benzoic acid, and 2-amino 3-hydroxy pyridine exhibit various biological activities such as antimicrobial activity, protein tyrosine phosphatases inhibition, and nuclease activity [2, 3]. Several aromatic amine Schiff bases have been investigated but few works deal with chromone skeleton derivatives. Chromones are a group of naturally occurring compounds that are ubiquitous in nature especially in plants. Molecules containing the chromone skeleton have extensive biological applications including antimycobacterial, antifungal, anticancer, antioxidant, antihypertensive, and anti-inflammatory applications and tyrosinase and protein kinase C inhibitors [4–13].

DNA plays an important role in the life process since it contains all the genetic information for the cellular function. However, DNA is the primary intracellular target of anticancer drugs, damaged under various conditions such as interactions with some small molecules, which cause DNA damage in cancer cells blocking the division of cancer cells and resulting in cell death. Metal complexes such as cobalt, nickel, copper, and zinc with Schiff base ligands have shown excellent binding and cleavage activities [14–16]. There is substantial literature supporting the DNA binding studies of chromone Schiff bases and their metal complexes [17–19]. Compounds showing the properties of effective binding as well as cleaving double stranded DNA under physiological conditions are of great importance since these could be used as diagnostic agents in medicinal and genomic research.

Fluorescent transition metal centres are particularly attractive moieties because they often possess distinctive electrochemical or photophysical properties, thus enhancing the functionality of the binding agent [20].

In previous papers we presented synthesis, detailed characterization, and biological activity studies of 3-formyl chromone Schiff bases such as 3-((2-hydroxy phenylimino)methyl)-4H-chromen-4-one (HL_1_), 2-((4-oxo-4H-chromen-3-yl)methylneamino)benzoic acid (HL_2_), 3-((3-hydroxypyridin-2-ylimino)methyl)-4H-chromen-4-one (HL_3_), 3-((2-mercapto phenylimino)methyl)-4H-chromen-4-one (HL_4_), and their Cu(II), Co(II), and Pd(II) complexes [21–23]. The present paper deals with the synthesis, characterization of Ni(II) and Zn(II) complexes of 3-formyl chromone Schiff bases (HL_1_, HL_2_, HL_3_, and HL_4_), and various biological activity studies, that is, antimicrobial, antioxidant, and nematicidal activities and cytotoxicity and DNA cleavage.

## 2. Experimental

### 2.1. Materials and Physical Measurements

All the chemicals used were of analytical grade and procured from Spectrochem Pvt. Ltd., Mumbai, India. 3-Formyl chromone was synthesized according to the literature [24].

The elemental analysis of carbon, hydrogen, nitrogen, and sulphur contents was performed using Perkin Elmer CHNS analyser. Molar conductance of the complexes was measured using a Digisun conductivity meter in DMF. The electronic absorption spectra of the complexes were recorded on JASCO V-670 Spectrophotometer in the wavelength region of 250–1400 nm in the solid state. The FTIR spectra of the complexes were recorded on Tensor 2 FTIR spectrophotometer in the region of 4000–400 cm^−1^ using KBr disc. The magnetic susceptibilities of Ni(II) complexes were measured with a Sherwood scientific balance. Diamagnetic corrections were calculated from Pascal's constants. The magnetic moment values were calculated using the relation *μ*_eff_ = 2.83  (*χ*_*m*_ *T*)^1/2^ B.M. Thermal studies of the complexes were carried out on a Perkin Elmer diamond TGA instrument at a heating rate of 10°C and nitrogen flow rate of 20 mL/min. The fluorescence spectra of the complexes were recorded on Fluorolog FL3-11 spectrofluorometer. The X-ray patterns of the complexes were recorded on Xpert-Pro X-ray diffractometer with Cu K*α* radiation (*λ* = 1.5406 Å). The diffraction data are integrated by using the Nakamuta program. Scanning electron micrograph (SEM) of the complexes was obtained in a Hitachi S-520 electron microscope at an accelerated voltage of 15 kV.

### 2.2. Synthesis of Ligands

**Figure 1:**
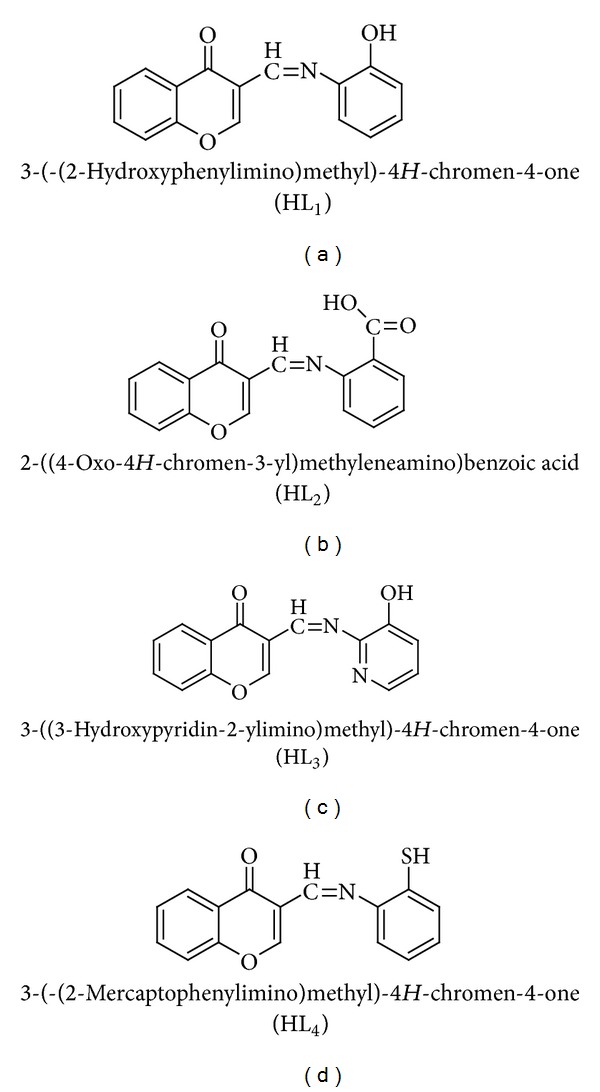
Structures of Schiff base ligands.

All the Schiff base ligands HL_1_, HL_2_, HL_3_, and HL_4_ (Figure 1) were prepared according to the literature methods [21, 25, 26].

### 2.3. Synthesis of Metal Complexes

The ligands (2 mM) and metal acetates (Ni(CH_3_COOH)_2_·4H_2_O and Zn(CH_3_COOH)_2_·2H_2_O) (1 mM) were dissolved in methanol. The mixtures were stirred at room temperature for 2-3 h; various colour precipitates were separated from the solution by suction filtration, purified by washing several times with methanol, and dried for 12 h in vacuum.

### 2.4. Antimicrobial Activity

*In vitro* antimicrobial activity of the metal complexes towards the bacteria* Proteus vulgaris, Klebsiella pnuemoniae, Staphylococcus aureus, *and* Bacillus subtili* and fungi* Candida albicans* was carried out using disc diffusion method. The antibiotics kanamycin and clotrimazole are the standards for antibacterial and antifungal activity studies. Standard inoculum, 1-2 × 10^7^ cfu/mL 0.5 Mc Farland standards [27], was introduced onto the surface of sterile nutrient agar plate and evenly spread by using a sterile glass spreader. Sterile antibiotic discs (6 mm in diameter, prepared using Whatmann number 1 paper) were placed over the nutrient agar medium. Each disc was spread by 100 *μ*g of the compounds (initially dissolved in DMSO). The plates were incubated with bacterial cultures for 24 h at 37°C and fungal cultures at 25°C for 48 h. The activity of the compounds was determined by measuring the diameter of inhibition zone in “millimetres” and compared with standard antibiotics. DMSO (which has no activity) and standard antibiotics were used as negative and positive controls for antimicrobial activity studies. The activity results are calculated as a mean of triplicates.

Minimum inhibitory concentrations (MIC) of the complexes which showed antimicrobial activity were determined using the literature method [28]. All the compounds that were diluted within the range of 100–10 *μ*g/mL were mixed in nutrient broth and 0.1 mL of active inoculums was added to each tube. The tubes were incubated aerobically at 37°C for bacteria and 25°C for fungi up to 24 h. The lowest concentration of the compound that completely inhibited bacterial growth (no turbidity) in comparison to control was regarded as MIC.

### 2.5. Nematicidal Activity

Root knot nematode,* Meloidogyne incognita*, is major plant parasitic nematodes affecting quantity and quality of the crop production in many annual and perennial crops.* Meloidogyne* nematode can develop galls and lesions in the roots, thereby causing stunted growth of the plants. Some of the chemicals can be used to control nematodes [29].

Nematicidal activity of the complexes was carried out on* Meloidogyne incognita*. Fresh egg masses of* Meloidogyne incognita* are collected from stock culture maintained on tomato (*Lycopersicon esculentum*) root tissues and kept in water for egg hatching. The eggs suspensions were poured on a cotton wool filter paper and incubated at 30°C to obtain freshly hatched juveniles (J2). Juveniles collected within 48 h were used for screening nematicidal activity of the compounds.

The compounds were initially dissolved in dimethyl sulfoxide (DMSO) and then in distilled water to make dilutions of 250, 150, and 50 *μ*g/mL. Experiments were performed under laboratory conditions at 30°C. About 100 freshly hatched second stage juveniles were suspended in 5 mL of each diluted compound and incubated. Distilled water with nematode larvae was taken as control. The dead nematodes were observed under an inverted binocular microscope. After an incubation of 24 and 48 h, percentage of mortality was calculated. Nematodes were considered dead if they did not move when probed with a fine needle [30].

### 2.6. DPPH Radical Scavenging Activity

The free radical scavenging activities of the metal complexes were determined by using DPPH free radical scavenging method according to the literature [31]. DPPH is a stable free radical containing an odd electron in its structure and usually utilized for detection of the radical scavenging activity in chemical analysis. In the spectrophotometric assay the ability to scavenge the stable free radical DPPH is measured by decrease in the absorbance at 517 nm. Each compound was dissolved in methanol (10 mg/10 mL) and it was used as stock solution. From the stock solutions, 1 mL of each compound solution with different concentrations (0.25 *μ*g–1.00 *μ*g) was added to the 3 mL of methanolic DPPH (0.004%) solution. After 30 min, the absorbance of the test compounds was taken at 517 nm using UV-VIS spectrophotometer. BHT was used as standard, DPPH solution was used as control without the test compounds, and methanol was used as blank. The percentage of scavenging activity of DPPH free radical was measured by using the following formula:

(1) $$\begin{array}{c} \text{Scavenging}\;\text{activity}\;\left(\%\right)=\left[\frac{\left(A_{o}-A_{i}\right)}{A_{o}}\right]\times 100, \end{array}$$

where *A*_*o*_ is the absorbance of the control and *A*_*i*_ is the absorbance of the sample.

### 2.7. Cytotoxic Activity

The human breast carcinoma cell line (MCF-7), human colon carcinoma cell line (COLO 205), and murine microphage cell line (Raw 264.7) were obtained from the National Centre for Cell Science (NCCS), Pune, and grown in Dulbecco's Modified Eagles Medium (DMEM) containing 10% fetal bovine serum (FBS), amphotericin (3 *μ*g/mL), gentamycin (400 *μ*g/mL), streptomycin (250 *μ*g/mL), and penicillin (250 units/mL) in a carbon dioxide incubator at 5% CO_2_. About 700 cells/well were seeded in 96-well plate using culture medium; the viability was tested using trypan blue dye with help of haemocytometer and 95% of viability was confirmed. After 24 h, the new medium with compounds in the concentration of 100, 10, and 1 *μ*g/mL were added at respective wells and kept in incubation for 48 h. After incubation MTT assay was performed.

#### 2.7.1. MTT Assay

After 48 h of the drug treatment the medium was changed again for all groups and 10 *μ*L of MTT (5 mg/mL stock solution) was added and the plates were incubated for an additional 4 h. The medium was discarded and the formazan blue, which was formed in the cells, was dissolved with 50 *μ*L of DMSO. The optical density was measured on microplate spectrophotometer at a wavelength of 570 nm. The percentage of cell inhibition was calculated by using the following formula [32]:

(2) $$\begin{array}{c} \%\text{Growth}\;\text{inhibition}=100-\left(\frac{A_{i}}{A_{o}}\right)\times 100, \end{array}$$

where *A*_*i*_ is the absorbance of the sample and *A*_*o*_ is the absorbance of the control. IC_50_ values were determined using Graph Pad Prism software.

### 2.8. DNA Cleavage Activity

The DNA cleavage activity of metal complexes was monitored by agarose gel electrophoresis. pUC19 plasmid was cultured, isolated, and used as DNA for the experiment. Test samples (1 mg/mL) were prepared in DMF. 25 *μ*g of the test samples was added to the isolated plasmid and incubated for 2 h at 37°C. After incubation, 30 *μ*L of plasmid DNA sample mixed with bromophenol blue dye (1 : 1) was loaded into the electrophoresis chamber wells along with the control DNA, 5 M FeSO_4_ (treated with DNA), and standard DNA marker containing TAE buffer (4.84 g Tris base, pH 8.0, 0.5 M EDTA/1 L). Finally, it was loaded on to an agarose gel and electrophoresed at 50 V constant voltage up to 30 min. After the run, gel was removed and stained with 10.01 *μ*g/mL ethidium bromide and image was taken in Versadoc (Biorad) imaging system. The results were compared with standard DNA marker. The same procedure was followed in the presence of H_2_O_2_ also.

## 3. Results and Discussion

All the Ni(II) complexes were colored, stable, and nonhygroscopic in nature. The complexes are insoluble in common organic solvents but soluble in DMF and DMSO. The elemental analysis showed that the complexes have 1 : 2 stoichiometry of the type [M(L_1–4_)_2_]·*n*H_2_O, where L stands for singly deprotonated ligands. Molar conductance of the complexes was measured in DMF. The conductance values, which are presented in Table 1, indicate the nonelectrolytic nature of the complexes [33].

### 3.1. Determination of the Metal Content of the Complexes

Known amount (0.150 g) of complexes was decomposed with concentrated nitric acid. This process was repeated till the organic part of the complexes got completely lost. The excess nitric acid was expelled by evaporation with concentrated sulphuric acid. The Ni(II) and Zn(II) contents of the complexes were determined as per the procedure available in the literature [34].

### 3.2. FTIR Spectra

The FTIR spectra of the complexes are compared with those of the ligands in order to determine the coordination sites that may involve in chelation. The position and/or the intensities of bands are expected to be changed while the coordination. The most important IR bands of the metal complexes with probable assignments are given in Table 2. The Schiff base ligands showing a band around 1650–1620 cm^−1^ is assigned to the (C=O) group of the chromone system. Upon complexation the *ν*(C=O) group is shifted to 20–35 cm^−1^ lower wavenumber region [35]. The ligands show the most characteristic *ν*(C=N) bands in the region 1605–1563 cm^−1^. In the spectra of their corresponding metal complexes, this band appears at 25–45 cm^−1^ to lower wavenumber region indicating the coordination of the azomethine nitrogen atom to the metal ion [36]. A broad band appeared in HL_1_ and HL_3_ at 3241 and 3246 cm^−1^, respectively, and is attributed to the *ν*(O–H) group. The absence of the band in the spectra of their corresponding metal complexes is due to the involvement of oxygen atom of (OH) group coordination to the metal ion. In HL_2_ ligand, a strong band appeared at 1365 cm^−1^ and is due to the *ν*(C–O) of carboxylic group. In its metal complexes it is shifted to 18–47 cm^−1^ lower wavenumber region [37]. Since SH stretching frequency band is very weak, the peak corresponding to SH is not clearly observed in the case of HL_4_ ligand [38]. Presence of band around 3400 cm^−1^ in all the complexes is the indication of water molecules present in the complexes. The presence of oxygen and nitrogen in the coordination sphere is further confirmed by the presence of *ν*(M–N) and *ν*(M–O) bands at 400–600 cm^−1^ region. The FTIR results show that all Schiff base ligands act as tridentate chelating ligands.

### 3.3. Electronic Spectra and Magnetic Moments

**Figure 2:**
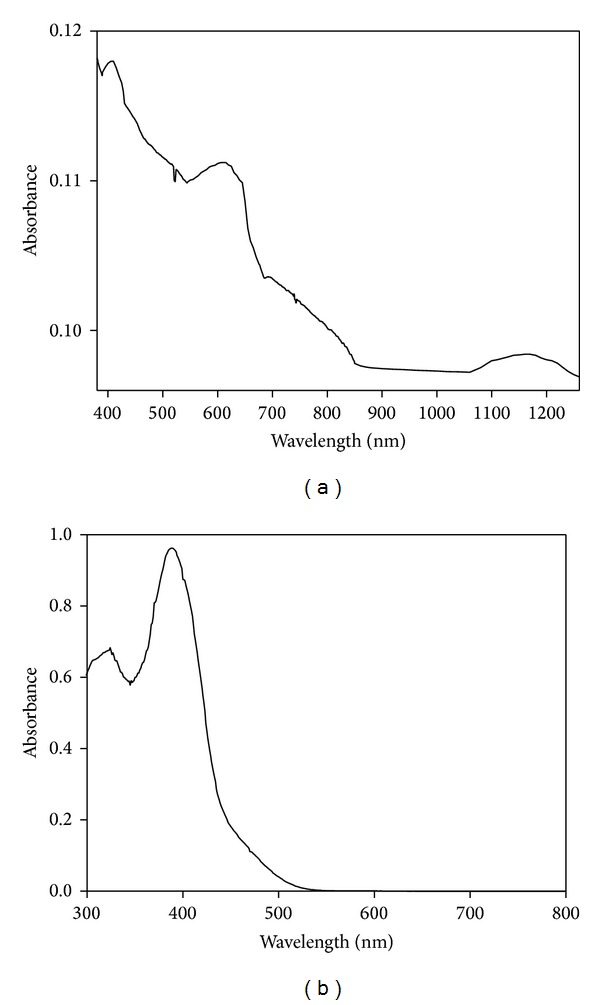
Electronic spectra of (a) [Ni(L_4_)_2_] and (b) [Zn(L_4_)_2_]·H_2_O.

The electronic absorption spectra of the Schiff base metal complexes in solid state were recorded at room temperature and the band positions of the absorption maxima, band assignments, ligand field parameters, and magnetic moment values are listed in Table 3. The electronic spectra of [Ni(L_4_)_2_]·H_2_O and [Zn(L_4_)_2_]·H_2_O complexes are depicted in Figure 2. The electronic spectra of Ni(II) complexes displayed three absorption bands in the range 8000–9000, 14000–16000, and 20000–24000 cm^−1^. Thus, these bands may be assigned to the three spin allowed transitions ^3^A_2g_ (F) →^3^T_2g_ (F) (*ν*_1_), ^3^A_2g_ (F) →^3^T_1g_ (F) (*ν*_2_), and ^3^A_2g_ (F) →^3^T_1g_ (P) (*ν*_3_), respectively, characteristic of octahedral geometry. The values of transition ratio *ν*_2_/*ν*_1_ and *β* lie in the range of 1.70–1.80 and 0.89–0.95, respectively, providing further evidence for octahedral geometry of Ni(II) complexes [39]. The B values for the complexes are lower than the free ion value, thereby indicating orbital overlap and delocalisation of d-orbitals. The *β*-values obtained are less than unity suggesting the covalent character of the metal-ligand bonds. All Ni(II) complexes are paramagnetic and the magnetic movement values at room temperature are in the range of 2.91–3.25 B.M which is well agreed with the reported octahedral Ni(II) complexes [40]. All Zn(II) complexes showed two bands around 25000 and 30000 cm^−1^ and are attributed to the *n* → π* and *π* → π* transitions, respectively. Zn(II) complexes that are in d^10^ configuration are diamagnetic and do not show any d-d transitions.

### 3.4. Thermal Analysis

**Figure 3:**
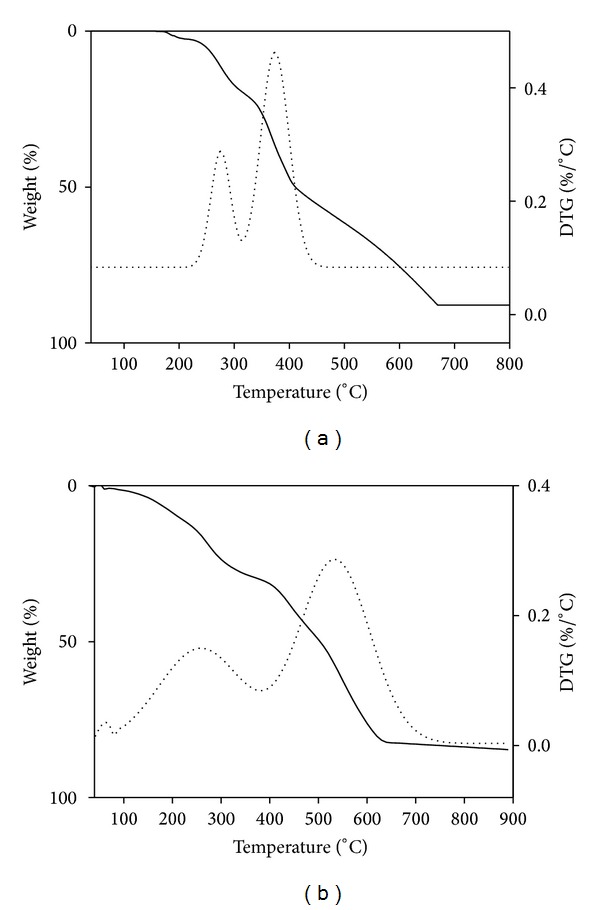
TG-DTG graph of (a) [Ni(L_4_)_2_] and (b) [Zn(L_4_)_2_]·H_2_O.

The thermogravimetric analysis (TGA) of the metal complexes was carried out within the temperature range from room temperature to 1000°C. The TG-DTA graphs of [Ni(L_4_)_2_] and [Zn(L_4_)_2_]·H_2_O complexes are given in Figure 3. The TGA data and their assignments of all the metal complexes are listed in Table 4. Metal complexes decompose gradually with formation of respective metal oxides. The TG graphs of [Ni(L_2_)_2_]·H_2_O, [Ni(L_3_)_2_]·H_2_O, [Zn(L_1_)_2_]·H_2_O, and [Zn(L_4_)_2_]·H_2_O complexes decompose in two to three steps. The first step corresponds to the loss of lattice water molecule in the temperature range between 30 and 140°C with a weight loss of 2-3%. In the second and third steps, the total loss of ligand molecules was observed in the temperature range between 160 and 850°C leaving behind metal oxide as residue. The thermal decomposition of remaining metal complexes ([Ni(L_1_)_2_], [Ni(L_4_)_2_] [Zn(L_2_)_2_] and [Zn(L_3_)_2_]) occurs in two to three steps. Upon starting heating, these metal complexes losses of the organic moieties of Schiff base ligands were observed in two to three successive steps within the temperature range of 150–850°C. In all the complexes the final mass loss is due to the formation of metal oxides as residue.

**Figure 4:**
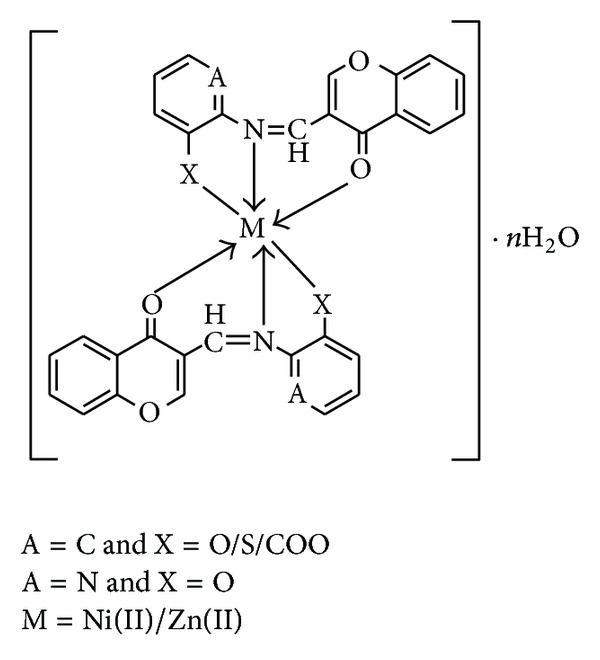
Proposed structure of the metal complexes.

On the basis of all spectral data (elemental analysis, FTIR, electronic spectra, and thermal analysis), the suggested structures of the complexes are shown in Figure 4.

### 3.5. Powder XRD and SEM Studies

**Figure 5:**
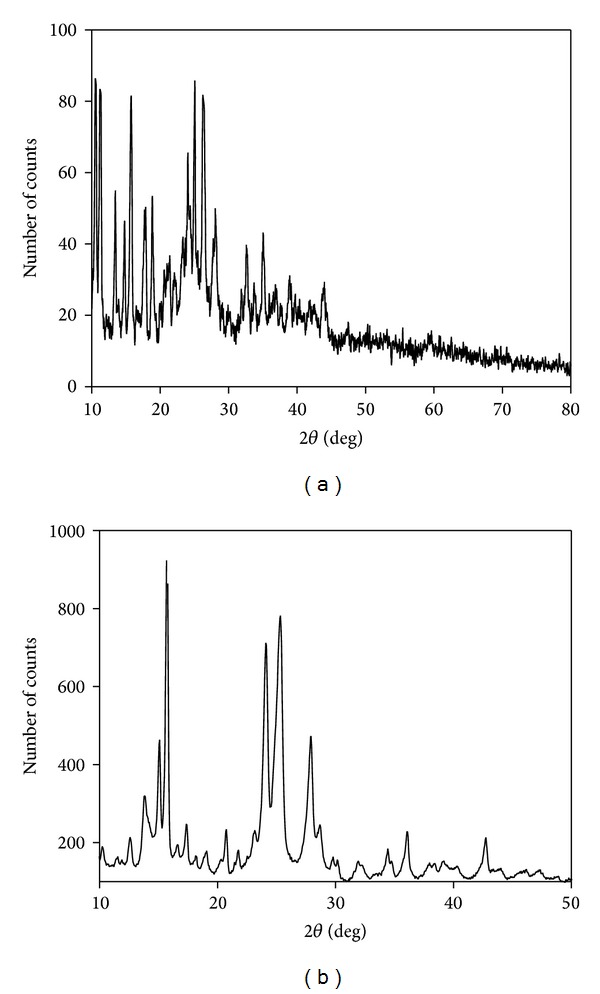
Powder XRD patterns of (a) [Ni(L_1_)_2_] (b) [Zn(L_1_)_2_]·H_2_O.

Single crystals of the complexes could not be obtained because of their insolubility in most organic solvents, hence, the powder diffraction data were obtained for structural characterization. The powder XRD patterns of all metal complexes were recorded in the range 2*θ* = 10–50°. The powder XRD pattern of [Ni(L_1_)_2_] and [Zn(L_1_)_2_]·H_2_O are presented in Figure 5. Observed and calculated powder XRD data of [Ni(L_1_)_2_] and [Zn(L_1_)_2_]·H_2_O are given in Tables 5(a) and 5(b). Unit cell parameters were found by using trial and error methods. All complexes are triclinic with different unit cell parameters. All metal complexes display sharp crystalline peaks except [Zn(L_2_)_2_] and [Zn(L_4_)_2_]·H_2_O; these two complexes do not exhibit well-defined crystalline peaks due to their amorphous nature. The unit cell parameters of metal complexes are as follows: [Ni(L_1_)_2_]: *a* = 8.80 Å, *b* = 8.52 Å, *c* = 7.50 Å, *α* = 99.93°, *β* = 116.71°, *γ* = 90.01°, and *V* = 562 (Å)^3^; [Ni(L_2_)_2_]·H_2_O: *a* = 8.24 Å, *b* = 9.24 Å, *c* = 7.32 Å, *α* = 104.12°, *β* = 124.44°, *γ* = 98.04°, and *V* = 557 (Å)^3^; [Ni(L_3_)_2_]·H_2_O: *a* = 9.40 Å, *b* = 9.20 Å, *c* = 8.46 Å, *α* = 97.45°, *β* = 107.38°, *γ* = 91.45°, and *V* = 731 (Å)^3^; [Ni(L_4_)_2_]: *a* = 7.73 Å, *b* = 8.24 Å, *c* = 8.49 Å, *α* = 98.30°, *β* = 104.76°, *γ* = 90.55°, and *V* = 540 (Å)^3^; [Zn(L_1_)_2_]·H_2_O: *a* = 9.60 Å, *b* = 9.94 Å, *c* = 8.89 Å, *α* = 102.34°, *β* = 109.69°, *γ* = 93.94°, and *V* = 848 (Å)^3^; [Zn(L_3_)_2_]: *a* = 8.76 Å, *b* = 9.04 Å, *c* = 8.61 Å, *α* = 105.78°, *β* = 103.02°, *γ* = 90.21°, and *V* = 682 (Å)^3^. The average crystallite sizes (*D*) of the metal complexes are calculated from the Scherrer formula [41]:

(3) $$\begin{array}{c} D=\frac{0.9\lambda }{\beta \cos\theta }, \end{array}$$

where *λ* is the X-ray wavelength, *β* is the full width at half maximum of prominent intensity peak, and *θ* is the diffraction angle. The [Ni(L_1_)_2_], [Ni(L_2_)_2_]·H_2_O, [Ni(L_3_)_2_]·H_2_O, [Ni(L_4_)_2_], [Zn(L_1_)_2_]·H_2_O, and [Zn(L_3_)_2_] complexes have an average crystallite size of 70, 17, 16, 27, 42, and 53 nm, respectively, suggesting that the complexes are in nanocrystalline phase.

**Figure 6:**
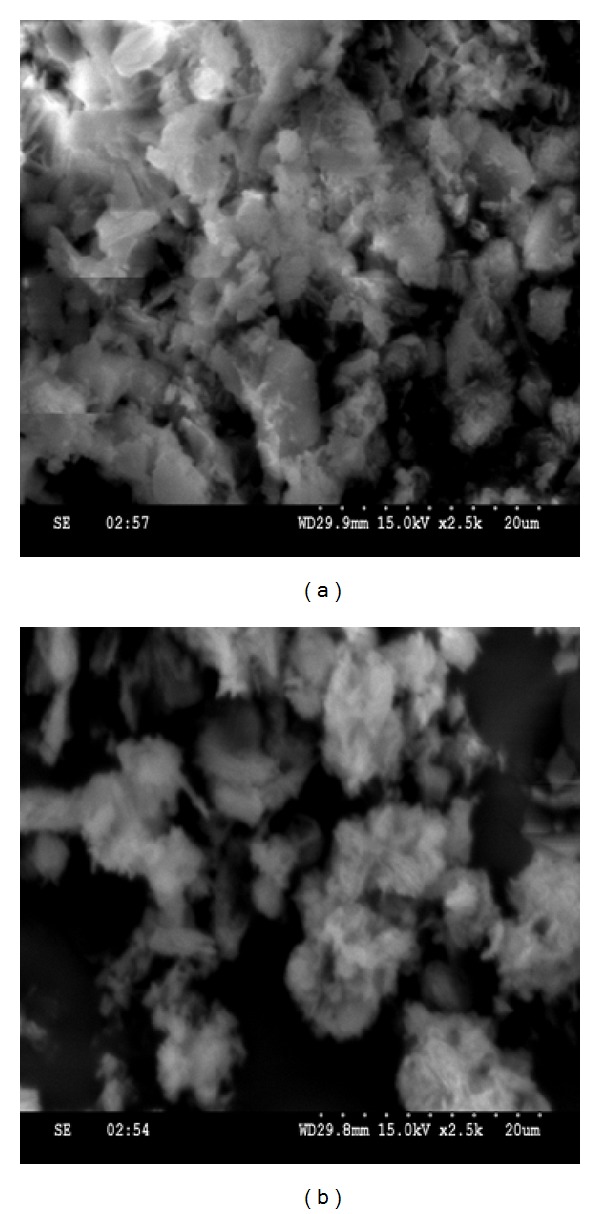
SEM micrograph of (a) [Ni(L_3_)_2_]·H_2_O and (b) [Ni(L_4_)_2_].

The SEM (Scanning Electron Microscope) is used to evaluate the morphology and particle size of the compounds. The SEM photographs of [Ni(L_3_)_2_]·H_2_O and [Ni(L_4_)_2_] are shown in Figure 6. The SEM micrographs show the agglomerate particles of the complexes. In case of [Ni(L_3_)_2_]·H_2_O and [Ni(L_4_)_2_] complexes, some agglomerates appear to have tiny needles, while the other agglomerates appear to be of spherical plates like morphologies.

### 3.6. Fluorescence Spectra

**Figure 7:**
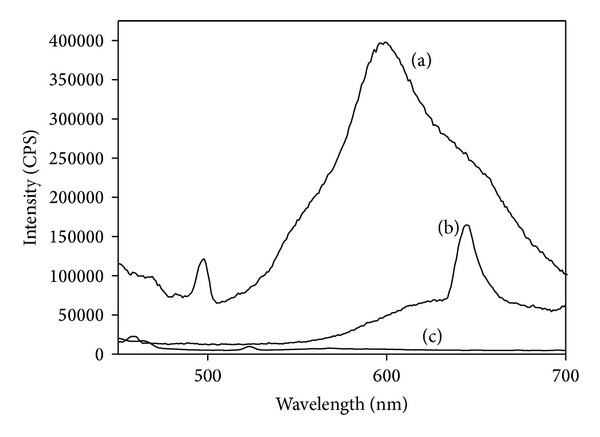
Fluorescence spectra of (a) HL_1_, (b) [Ni(L_1_)_2_], and (c) [Zn(L_1_)_2_]·H_2_O.

The fluorescence characteristics of metal complexes were studied at room temperature in solid state. In metal complexes metal to ligand coordination may lead to significant changes of the fluorescence properties of the ligand, including increase or decrease of the intensity, emission wavelength shift, quenching of the fluorescence, or appearance of new emissions [42]. The fluorescence spectra of HL_1_ ligand and its metal (Ni(II) and Zn(II)) complexes were depicted in Figure 7. The Ni(II) and Zn(II) complexes of HL_1_ were characterised by emission bands around (522, 569) nm and (458, 644) nm, upon photo excitation at 453 and 357 nm, respectively. The Ni(II) complex of HL_2_ exhibits emission bands at 449 and 524 nm. Zn(II) complex of HL_2_ does not show any emission spectra. The Ni(II) and Zn(II) complexes of HL_3_ were characterised by an emission bands at (504, 570, and 668) and 419 nm, respectively. The Ni(II) and Zn(II) complexes of HL_4_ exhibit emission bands around 539 and 613 nm, respectively. From the results, red shift and quenching of the metal ions was observed in the case of ligands and its metal complexes.

### 3.7. Antimicrobial Activity

The minimum inhibitory concentrations (MIC) of the complexes compared with the ligands and standard drugs are listed in Table 6. The results indicate that the metal complexes displayed more antibacterial activity compared to the parent ligands under similar experimental conditions on same microorganisms except [Ni(L_2_)_2_]·H_2_O and [Zn(L_1_)_2_]·H_2_O complexes. The results indicate that the metal complexes displayed more antibacterial activity compared to the parent ligands under similar experimental conditions on same microorganisms except [Zn(L_1_)_2_]·H_2_O. However, [Ni(L_1_)_2_], [Ni(L_3_)_2_]·H_2_O, [Zn(L_2_)_2_] and [Zn(L_3_)_2_] complexes showed antifungal activity while the remaining complexes did not show any activity. However, [Ni(L_1_)_2_], [Ni(L_3_)_2_]·H_2_O, and [Zn(L_3_)_2_] complexes showed antifungal activity while the remaining complexes did not show any activity. Among all the metal complexes [Zn(L_3_)_2_] complex showed good activity against all bacteria and fungi strains. Increase in the activity of the complexes compared to that of ligands can be explained on the basis of Overtone's concept and Tweedy's chelation theory. The theory states that the polarity of the metal ion is reduced on complexation due to the partial sharing of its positive charge with donor groups. Consequently, the positive charge is delocalized over the whole ring, which causes the improved lipophilicity of the compound through cell membrane of the pathogen [43]. The negative results can be attributed either to the inability of the complexes to diffuse into the bacteria cell membrane and hence they become unable to interfere with its biological activity or they can diffuse and become inactivated by unknown cellular mechanism, that is, bacterial enzymes [44].

### 3.8. Nematicidal Activity

All complexes except [Ni(L_3_)_2_]·H_2_O and [Zn(L_3_)_2_] showed very less nematicidal activity against* Meloidogyne incognita*. However, [Ni(L_3_)_2_]·H_2_O and [Zn(L_3_)_2_] complexes exhibited moderate activity indicating 47% and 51% mortality, respectively, after 48 h exposure in 250 *μ*g/mL concentration. However, the activity of the metal complexes depends on concentration and time, that is, activity was higher at high concentrations and increased with time.

### 3.9. DPPH Radical Scavenging Activity

The antioxidant activity of the complexes was tested by measuring their ability to donate an electron to the free radical compound DPPH, monitoring changes in absorption at 517 nm using UV-VIS spectrophotometer. IC_50_ values were calculated and compared with the standard. IC_50_ values of the metal complexes are tabulated in Table 7. All complexes showed very less activity except [Ni(L_1_)_2_], [Ni(L_4_)_2_], and [Zn(L_4_)_2_]·H_2_O complexes. Among all the complexes [Zn(L_4_)_2_]·H_2_O (IC_50_ = 0.69 *μ*g/mL) complex exhibited comparable activity to that of standard drug BHT (butylated hydroxyl toluene) (IC_50_ = 0.67 *μ*g/mL).

### 3.10. Cytotoxic Activity

The cytotoxicity of Schiff base ligands and their metal complexes were carried out using MTT assay. IC_50_ is employed to stand for the cytotoxicities of the compounds against the cancer cell lines; the smaller the IC_50_ value in the same condition is, the higher the cell growth inhibitory potency. DMSO when used as control without test sample, it does not exhibit any cytotoxic activity against all cancer cell lines. Cis-platin is used as positive control and it showed the highest cytotoxicity. The IC_50_ values were calculated after 48 h of incubation with compounds and are listed in Table 8. As shown in Table 8, all the ligands and their metal complexes showed lower cytotoxicity against selected cancer cell lines compared to the cis-platin. HL_3_ ligand showed enhanced activity against COLO 205 cell lines compared to all ligands and metal complexes (IC_50_ = 15.2 *μ*g/mL). The lowest IC_50_ values (33.1 and 22.6 *μ*g/mL) are observed for Zn(II) complex of HL_3_ ligand against raw and MCF-7 cell lines among all the ligands and metal complexes indicating its significant potency against two cancer cell lines. The results revealed that pyridine ring may be playing an important role in the cytotoxicity of the compounds.

### 3.11. DNA Cleavage Activity

**Figure 8:**
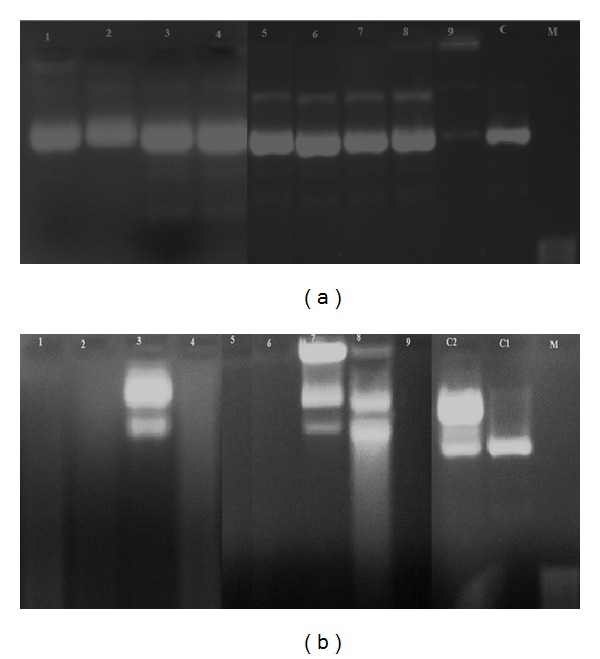
Gel electrophoresis photograph of metal complexes. (a) Gel electrophoresis photograph showing the effects of metal complexes on pUC 19 DNA: lane 1, DNA + [Ni(L_1_)_2_]; lane 2, DNA + [Ni(L_2_)_2_]·H_2_O; lane 3, DNA + [Ni(L_3_)_2_]·H_2_O; lane 4, DNA + [Ni(L_4_)_2_]; lane 5, DNA + [Zn(L_1_)_2_]·H_2_O; lane 6, DNA + [Zn(L_2_)_2_]; lane 7, DNA + [Zn(L_3_)_2_]; lane 8, DNA + [Zn(L_4_)_2_]·H_2_O; lane 9, DNA + FeSO_4_; lane C, DNA alone. (b) Gel electrophoresis photograph showing the effects of metal complexes on pUC 19 DNA in the presence of H_2_O_2_: lane 1, DNA + [Ni(L_1_)_2_] + H_2_O_2_; lane 2, DNA + [Ni(L_2_)_2_]·H_2_O + H_2_O_2_; lane 3, DNA + [Ni(L_3_)_2_]·H_2_O + H_2_O_2_; lane 4, DNA + [Ni(L_4_)_2_] + H_2_O_2_; lane 5, DNA + [Zn(L_1_)_2_]·H_2_O + H_2_O_2_; lane 6, DNA + [Zn(L_2_)_2_] + H_2_O_2_; lane 7, DNA + [Zn(L_3_)_2_] + H_2_O_2_; lane 8, DNA + [Zn(L_4_)_2_]·H_2_O + H_2_O_2_; lane 9, DNA + FeSO_4_ + H_2_O_2_; lane C2, DNA + H_2_O_2_; lane C1, DNA alone.

pUC19 DNA was chosen for the DNA cleavage activity of the complexes. The studies were done using gel electrophoresis. Its naturally occurring supercoiled form (Form I) when nicked gives rise to an open circular relaxed form (Form II) and upon further cleavage results in the linear form (Form III). When subjected to gel electrophoresis, Form I migrates relatively faster while the nicked form (Form II) migrates slowly and linearized Form III migrates between Form I and Form II [45]. In the present study gel electrophoresis experiments were performed in the presence and absence of an oxidizing agent H_2_O_2_ to investigate the mechanism of nucleolytic activity of the complexes. The activity was greater for the complexes in the presence of H_2_O_2_. Control (DNA alone) does not show activity (Figure 8(a)). When FeSO_4_ is used as standard, it shows complete DNA cleavage. In the absence of H_2_O_2_ all complexes show partial nucleolytic activity. Probably this may be due to the redox behaviour of the metal ions. These results indicated the important role of the metal ions in cleavage studies. In the presence of H_2_O_2_, (Figure 8(b)) absence of marker bands was observed in all the complexes except [Ni(L_3_)_2_]·H_2_O and [Zn(L_3_)_2_]; [Zn(L_4_)_2_]·H_2_O complexes indicate the complete DNA cleavage activity. In the case of [Ni(L_3_)_2_]·H_2_O, [Zn(L_3_)_2_], and [Zn(L_4_)_2_]·H_2_O complexes a decrease in the intensity of bands was observed compared to the control. This is probably due to the partial cleavage of the DNA. The DNA cleavage activity of the complexes in the presence of H_2_O_2_ may be due to the reaction of hydroxy radicals with DNA. The general oxidative mechanisms of the DNA cleavage studies were reported by several research groups [46–48]. Many literature reports infer that the compound was to cleave the DNA; it can be concluded that the compound inhibits the growth of the pathogenic organism by cleaving the genome [49].

## 4. Conclusions

Ni(II) and Zn(II) complexes have been synthesized using 3-formyl chromone Schiff bases and characterized by various analytical and spectral data. Based on the electronic spectra, magnetic moment, and elemental analysis data, octahedral geometry was proposed for Ni(II) and Zn(II) complexes. The well-defined crystalline and homogeneous nature of the metal complexes is observed from powder XRD and SEM analyses. The antimicrobial activity data has shown that [Zn(L_3_)_2_] complex displayed higher activity among all other metal complexes. The nematicidal activity of metal complexes revealed that [Ni(L_3_)_2_]·H_2_O and [Zn(L_3_)_2_] complexes showed moderate activity. [Zn(L_4_)_2_]·H_2_O complex exhibited greater antioxidant activity compared to the remaining metal complexes. All metal complexes exhibited considerable cytotoxic activity against Raw, MCF-7, and COLO 205 cell lines. The DNA cleavage studies of metal complexes showed more prominent activity in the presence of H_2_O_2_ compared to that in the absence of H_2_O_2_.

## Figures and Tables

**Table 1:** Elemental analysis and physical properties of metal complexes.

Molecular formula	Colour (% yield)	% found (cald.)					Molar conductivity (Ohm^−1^ cm^2^ mol^−1^)
		C	H	N	S	M (Ni/Zn)	
[Ni(L_1_)_2_]	Brick red	64.49	3.21	4.23		9.91	10
[Ni(C_16_H_10_O_3_N)_2_]	(71)	(65.45)	(3.40)	(4.77)		(10.00)	
[Ni(L_2_)_2_]·H_2_O	Light green	61.59	3.02	4.21		8.79	12
[Ni(C_17_H_10_O_4_N)_2_]·H_2_O	(62)	(61.75)	(3.32)	(4.24)		(8.88)	
[Ni(L_3_)_2_]·H_2_O	Brick red	61.07	3.23	9.23		9.43	15
[Ni(C_15_H_9_O_3_N_2_)_2_]·H_2_O	(85)	(61.15)	(3.39)	(9.51)		(9.97)	
[Ni(L_4_)_2_]	Black	62.21	3.09	4.61	9.30	9.21	14
[Ni(C_16_H_10_O_2_NS)_2_]	(82)	(62.06)	(3.23)	(4.52)	(10.34)	(9.49)	
[Zn(L_1_)_2_]·H_2_O	Orange	62.91	3.52	4.62		10.58	15
[Zn(C_16_H_10_O_3_N)_2_]·H_2_O	(88)	(62.80)	(3.60)	(4.58)		(10.70)	
[Zn(L_2_)_2_]	Yellow	62.79	3.15	4.25		9.95	11
[Zn(C_17_H_10_O_4_N)_2_]	(61)	(62.83)	(3.08)	(4.31)		(10.07)	
[Zn(L_3_)_2_]	Brick red	60.21	3.25	9.35		10.51	18
[Zn(C_15_H_9_O_3_N_2_)_2_]	(87)	(60.46)	(3.02)	(9.40)		(10.98)	
[Zn(L_4_)_2_]·H_2_O	Yellow	60.22	3.35	4.21	10.01	10.31	20
[Zn(C_16_H_10_O_2_NS)_2_]·H_2_O	(73)	(59.68)	(3.41)	(4.35)	(9.93)	(10.46)	

**Table 2:** IR spectral data of ligands and their metal complexes (cm^−1^).

Compound	*ν*(OH)	*ν*(C=O) (*γ* pyrone)	*ν*(C=N)	*ν*(CO) (carboxylate)	*ν*(C–S)	*ν*(M–N)	*ν*(M–O)
HL_1_	3241	1643	1605				
HL_2_		1651	1605	1365			
HL_3_	3246	1647	1591				
HL_4_		1622	1597		790		
[Ni(L_1_)_2_]		1619	1563			467	586
[Ni(L_2_)_2_]·H_2_O		1618	1570	1318		419	514
[Ni(L_3_)_2_]·H_2_O		1615	1563			480	500
[Ni(L_4_)_2_]		1598	1564		743	443	524
[Zn(L_1_)_2_]·H_2_O		1614	1574			460	599
[Zn(L_2_)_2_]		1615	1563	1347		480	500
[Zn(L_3_)_2_]		1615	1563			488	521
[Zn(L_4_)_2_]·H_2_O		1609	1581		751	441	505

**Table 3:** Electronic, magnetic, and ligand field parameters of Ni(II) complexes.

Compound	Absorption maxima (cm^−1^)	Tentative assignments	Magnetic moment (B.M)	*ν*_2_/*ν*_1_	10 Dq (cm^−1^)	B (cm^−1^)	*β*	LFSE (kJ·mol^−1^)
[Ni(L_1_)_2_]	8532 15200 24900	^ 3^A_2g_(F) → ^3^T_2g_(F) (*ν*_1_) ^3^A_2g_(F) → ^3^T_1g_(F) (*ν*_2_) ^3^A_2g_(F) → ^3^T_1g_(P) (*ν*_3_)	3.12	1.78	8532	966	0.92	122.50

[Ni( L_2_)_2_]·H_2_O	8787 16000 24390	^ 3^A_2g_(F) → ^3^T_2g_(F) (*ν*_1_) ^3^A_2g_(F) → ^3^T_1g_(F) (*ν*_2_) ^3^A_2g_(F) → ^3^T_1g_(P) (*ν*_3_)	3.18	1.82	8787	935	0.89	126.19

[Ni(L_3_)_2_]·H_2_O	8313 14705 20833 24390	^ 3^A_2g_(F) → ^3^T_2g_(F) (*ν*_1_) ^3^A_2g_(F) → ^3^T_1g_(F) (*ν*_2_) ^3^A_2g_(F) → ^3^T_1g_(P) (*ν*_3_)	3.25	1.76	8313	943	0.90	119.40

[Ni(L_4_)_2_]	8628 16313 24570	^ 3^A_2g_(F) → ^3^T_2g_(F) (*ν*_1_) ^3^A_2g_(F) → ^3^T_1g_(F) (*ν*_2_) ^3^A_2g_(F) → ^3^T_1g_(P) (*ν*_3_)	2.91	1.89	8628	999	0.95	123.92

**Table 4:** Thermal analysis results of metal complexes.

Compound	Temperature (°C)	Found (cald.)	Assignment
[Ni(L_1_)_2_] [Ni(C_16_H_10_O_3_N)_2_]	180–420	19.38 (18.36)	C_6_H_4_ON
	421–718	68.55 (69.02)	C_26_H_16_O_5_N
	>718	12.07 (12.62)	NiO

[Ni(L_2_)_2_]·H_2_O [Ni(C_17_H_10_O_4_N)_2_]·H_2_O	30–90	2.83 (2.72)	H_2_O
	335–478	60.71 (62.05)	C_24_H_14_O_5_N_2_
	479–849	23.93 (23.61)	C_10_H_6_O_2_
	>849	12.53 (11.62)	NiO

[Ni(L_3_)_2_]·H_2_O [Ni(C_15_H_9_O_3_N_2_)_2_]·H_2_O	30–65	3.09 (2.96)	H_2_O
	180–879	83.40 (84.73)	C_30_H_18_O_5_N_4_
	>880	13.51 (12.31)	NiO

[Ni(L_4_)_2_] [Ni(C_16_H_10_O_2_NS)_2_]	171–410	45.91 (45.25)	C_16_H_10_O_2_NS
	411–670	42.14 (42.67)	C_16_H_10_ONS
	>670	11.95 (12.07)	NiO

[Zn(L_1_)_2_]·H_2_O [Zn(C_16_H_10_O_3_N)_2_]·H_2_O	30–135	2.45 (2.95)	H_2_O
	194–422	24.95 (25.92)	C_10_H_6_O_2_
	423–790	59.48 (58.09)	C_22_H_14_O_3_N_2_
	>791	13.12 (13.04)	ZnO

[Zn(L_2_)_2_] [Zn(C_17_H_10_O_4_N)_2_]	195–265	15.55 (16.01)	C_7_H_4_O
	266–661	69.76 (71.46)	C_27_H_16_O_4_N_2_
	>662	14.69 (12.53)	ZnO

[Zn(L_3_)_2_] [Zn(C_15_H_9_O_3_N_2_)_2_]	157–192	10.91 (12.93)	C_5_H_3_N
	193–485	28.65 (29.22)	C_10_H_6_O_2_N
	486–835	46.03 (44.18)	C_15_H_9_O_3_N_2_
	>836	14.41 (13.67)	ZnO

[Zn(L_4_)_2_]·H_2_O [Zn(C_16_H_10_O_2_NS)_2_]·H_2_O	30–124	2.41 (2.79)	H_2_O
	165–348	27.41 (26.73)	C_10_H_6_O_2_N
	389–632	56.98 (57.84)	C_22_H_14_ONS_2_
	>632	13.20 (12.64)	ZnO

**Table:** (a)

S. number	2*θ*		Δ2*θ*	*d*-spacing		*h*	*k*	*l*
	Observed	Calculated		Observed	Calculated			
1	10.57	10.57	0	8.3627	8.3627	0	1	0
2	11.29	11.29	0	7.8314	7.8314	1	0	0
3	13.44	13.44	0	6.5805	6.5805	0	0	1
4	14.79	14.79	0	5.9847	5.9847	−1	1	0
5	15.75	15.75	0	5.6205	5.6205	−1	−1	1
6	17.79	17.79	0	4.9805	4.9805	−1	1	1
7	18.85	18.68	0.164	4.7046	4.7455	0	1	1
8	21.16	21.22	−0.06	4.1962	4.1845	1	0	1
9	22.14	22.06	0.074	4.0121	4.0255	1	−1	1
10	24.05	24.07	−0.013	3.6968	3.6948	−1	0	2
11	25.04	24.99	0.054	3.5533	3.5608	1	2	0
12	26.33	26.35	−0.017	3.382	3.3799	−1	2	1
13	28.1	28.04	0.061	3.1731	3.1799	−1	1	2
14	32.65	32.65	0	2.7404	2.7405	2	2	0
15	35.06	35.07	−0.012	2.5575	2.5566	−3	1	0
16	38.92	38.86	0.054	2.3124	2.3155	−2	3	1
17	43.89	43.95	−0.058	2.0612	2.0586	−1	3	2
18	45.13	45.16	−0.033	2.0076	2.0062	−2	−3	3

**Table:** (b)

S. number	2*θ* values		Δ2*θ*	*d*-spacing		*h*	*k*	*l*
	Observed	Calculated		Observed	Calculated			
1	12.58	12.58	0	7.0315	7.0315	1	1	0
2	13.81	13.81	0	6.4077	6.4077	−1	0	1
3	15.73	15.73	0	5.6299	5.6299	1	0	1
4	17.35	17.35	0	5.1064	5.1064	−1	1	1
5	20.75	20.77	−0.017	4.2777	4.2742	−1	3	0
6	24.09	24.13	−0.031	3.6907	3.6859	−1	−3	2
7	25.32	25.31	0.012	3.5141	3.5158	2	2	0
8	27.93	27.97	−0.038	3.1916	3.1873	−1	−4	2
9	30.15	30.16	−0.008	2.9620	2.9612	0	−3	3
10	31.92	31.90	0.021	2.8015	2.8033	2	−5	2
11	34.43	34.45	−0.017	2.6028	2.6016	−1	5	0
12	36.05	36.06	−0.008	2.4892	2.4886	−1	0	3
13	42.75	42.83	−0.081	2.1137	2.1099	3	1	2

**Table 6:** MIC values of antimicrobial activity of ligands and their metal complexes (*µ*g/mL).

Compound	*Bacillus subtilis *	*Staphylococcus aureus *	*Proteus vulgaris *	*Klebsiella pneumoniae *	*Candida albicans *
HL_1_	—	—	—	—	—
HL_2_	80	80	80	80	80
HL_3_	—	—	—	—	—
HL_4_	—	—	—	—	—
[Ni(L_1_)_2_]	35	40	32	41	75
[Ni(L_2_)_2_]·H_2_O	70	80	65	85	—
[Ni(L_3_)_2_]·H_2_O	45	45	50	33	33
[Ni(L_4_)_2_]	75	80	—	78	—
[Zn(L_1_)_2_]·H_2_O	—	—	—	—	—
[Zn(L_2_)_2_]	85	78	80	75	80
[Zn(L_3_)_2_]	30	30	33	30	33
[Zn(L_4_)_2_]·H_2_O	—	80	—	80	—
Kanamycin	4	10	8	11	—
Clotrimazole	—	—	—	—	10

**Table 7:** IC_50_ values (*µ*g/mL) of DPPH radical scavenging activity of ligands and their metal complexes.

Compound	IC_50_ (*µ*g/mL)
HL_3_	1.27
HL_4_	0.40
[Ni(L_1_)_2_]	1.21
[Ni(L_4_)_2_]	1.09
[Zn(L_4_)_2_]·H_2_O	0.69
BHT	0.67

**Table 8:** IC_50_ values (*µ*g/mL) of cytotoxic activity of ligands and their metal complexes.

Compound	Raw	MCF-7	COLO 205
HL_1_	46.8	24.5	20.1
HL_2_	56.1	34.2	39.1
HL_3_	52.8	29.2	15.2
HL_4_	46.9	30.4	68.1
[Ni(L_1_)_2_]	224.8	67.1	54.6
[Ni( L_2_)_2_]·H_2_O	36.3	26.5	60.9
[Ni(L_3_)_2_]·H_2_O	53.0	36.9	71.4
[Ni(L_4_)_2_]	58.6	35.6	49.9
[Zn(L_1_)_2_]·H_2_O	44.0	40.1	54.7
[Zn(L_2_)_2_]	34.0	52.3	43.9
[Zn(L_3_)_2_]	33.1	22.6	42.0
[Zn(L_4_)_2_]·H_2_O	42.5	39.2	53.5
Cis-platin	1.5	1.7	5.6
